# A Case of Stereotactic Body Radiotherapy for Inoperable Intraductal Papillary Neoplasm of the Bile Duct

**DOI:** 10.7759/cureus.80916

**Published:** 2025-03-20

**Authors:** Masayoshi Inoue, Emiko Shimoda, Shota Rikihisa, Fumiaki Isohashi

**Affiliations:** 1 Department of Radiology, Higashiosaka City Medical Center, Higashiosaka, JPN; 2 Department of Radiation Oncology, Nara Medical University, Kashihara, JPN

**Keywords:** cholangiltis, endoscopic retrograde cholangiopancreatography (ercp), intraductal papillary neoplasms of the bile duct (ipnb), mucin production, peroral cholangioscopy, stereotactic body radiotherapy

## Abstract

Intraductal papillary neoplasms of the bile duct (IPNBs) have a high propensity for malignant transformation; therefore, surgical resection remains the standard first-line treatment. In cases where hepatic lobectomy is not feasible, however, controlling biliary drainage can be challenging. We present herein a case of unresectable IPNB complicated by jaundice and cholangitis due to mucin production by the tumor. Stereotactic body radiation therapy (SBRT) has been used as a treatment modality, resulting in favorable outcomes. An 80-year-old male patient presented to the emergency room with symptoms of cholangitis, for which he had previously undergone endoscopic lithotripsy for the removal of choledochal stones. The persistent symptoms and viscous bile led to suspicions of IPNB; therefore, the patient underwent a peroral cholangioscopy with biopsy to confirm the diagnosis. Due to his compromised cardiac function, however, surgical resection was not deemed a viable option, and biliary drainage was attempted instead. Unfortunately, frequent mucin-induced obstructions of the catheter made it difficult to control the cholangitis; therefore, SBRT was administered to achieve local tumor control and reduce mucin production. The radiation was delivered using a 10 MV beam with a volumetric-modulated arc therapy, fiducial gold markers, and suspended exhalation. A total dose of 60 Gy was administered across eight fractions (planning target volume D95), and one month post-SBRT, the biliary dilation and cholangitis had resolved. No local tumor regrowth or recurrent cholangitis occurred for at least one year after treatment, nor were any significant adverse effects related to the SBRT observed. According to the Surveillance, Epidemiology, and End Results (SEER) database, SBRT has been performed for IPNB; however, only a few case reports have been identified, all of which were cases in which brachytherapy was performed. To our knowledge, this is the first case report detailing a treatment regimen of SBRT with a definite radiation dose. This case suggests that SBRT may be of value as an alternative therapy for cases in which surgical resection is not an option.

## Introduction

Intraductal papillary neoplasms of the bile duct (IPNBs) are rare tumors located within the bile duct, characterized by papillary or villous growths covered with neoplastic epithelium. These tumors typically exhibit fine fibrovascular stalks within dilated bile ducts [1,2], have been reported to occur predominantly in East Asia [3], and are associated with a relatively high rate of malignancy at initial diagnosis as well as a high potential for malignant transformation [4,5]. IPNB, therefore, is treated as a precursor lesion to invasive cholangiocarcinoma, and surgical intervention is the standard first-line treatment for all eligible cases. However, as surgical interventions such as hepatic lobectomy and bile duct resection are highly invasive, the number of eligible cases is limited, and when surgical resection is not a viable option, the management of obstructive jaundice and cholangitis due to mucin production is often a significant challenge. According to an observational study using the Surveillance, Epidemiology, and End Results (SEER) database, nonsurgical treatments primarily consist of postoperative pharmacotherapy, with additional implementation of pharmacotherapy and radiation therapy for unresectable cases [4]. However, the approaches vary widely, ranging from curative-intent therapy aimed at prolonging survival to palliative care, and no consensus has been established. In particular, radiation therapy has predominantly been used for palliative purposes [6,7]. Although external-beam radiation therapy, including brachytherapy, has been used, to the best of our knowledge, no reports have documented stereotactic body radiation therapy (SBRT) with respiratory motion management delivering a curative dose for IPNB. In this report, we present a case of unresectable IPNB with mucin production treated with SBRT, achieving a favorable therapeutic outcome. We further discuss this case in the context of a literature review.

## Case presentation

**Figure 1:**
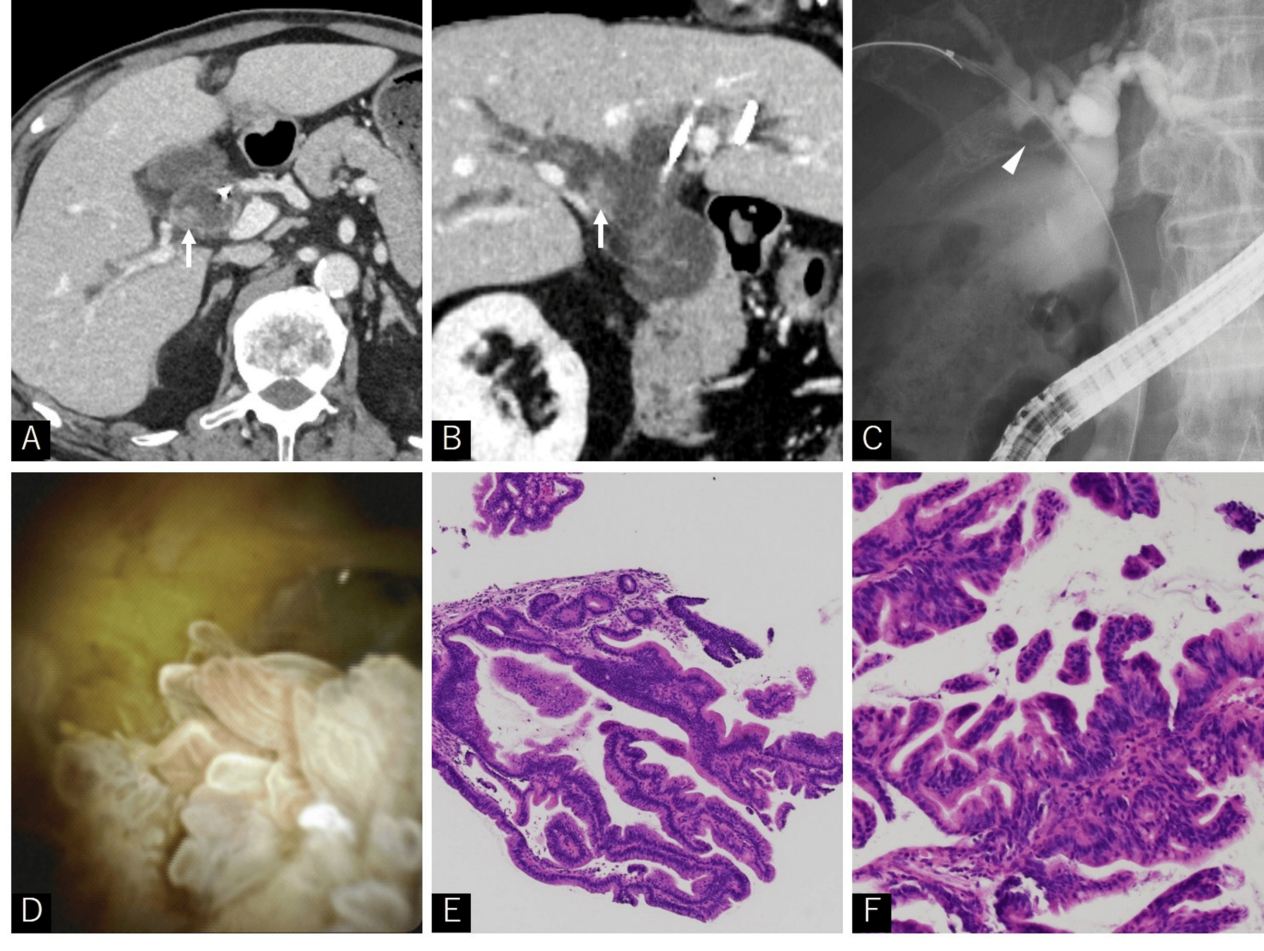
Radiological findings and pathological features of intraductal papillary neoplasm of the bile duct Enhanced computed tomography (CT) axial view (A) and coronal view (B) demonstrated dilatation of the intrahepatic and common bile ducts, and an enhancing nodule was detected within the first branch of the right hepatic duct (arrow). Endoscopic retrograde cholangiopancreatography showed a filling defect in the first branch of the right hepatic duct (arrowhead), suggesting a possible tumor demonstrated on the enhanced CT (C). Peroral cholangioscopy (PCOS) revealed white papillary elevations with mucin production in the first branch of the right hepatic duct (D). Intraductal papillary neoplasm of the bile duct (IPNB) with low-grade intraepithelial neoplasia (E: magnification, ×40 and F: magnification, ×100; hematoxylin and eosin staining)

An 80-year-old male patient with a history of percutaneous coronary intervention for myocardial infarction presented to the emergency department with a chief complaint of right upper abdominal pain, which he experienced during a follow-up visit with cardiology. A hematological evaluation revealed elevated serum bilirubin levels, while sonography of the abdomen showed dilation of the intrahepatic and common bile ducts. The patient underwent magnetic resonance cholangiopancreatography (MRCP), which revealed filling defects in the lower common bile duct and proximal right hepatic duct, suggesting the presence of gallstones. At this point, IPNB was not suspected, and the patient was diagnosed with obstructive jaundice due to common bile duct stones, accompanied by cholangitis. Therefore, endoscopic retrograde cholangiopancreatography (ERCP) was performed to remove the stones, after which the patient’s symptoms were relieved. As the dilation of the bile duct was not improved one month post-procedure, however, another ERCP was performed, which revealed mucin secretion from within the bile duct into the duodenum via the papilla of Vater, suggestive of IPNB. Subsequent peroral cholangioscopy revealed a papillary tumor in the hilum of the right hepatic duct. The tumor was biopsied, leading to a diagnosis of type 1 IPNB with low-to-moderate dysplasia (Figure 1).

**Figure 2:**
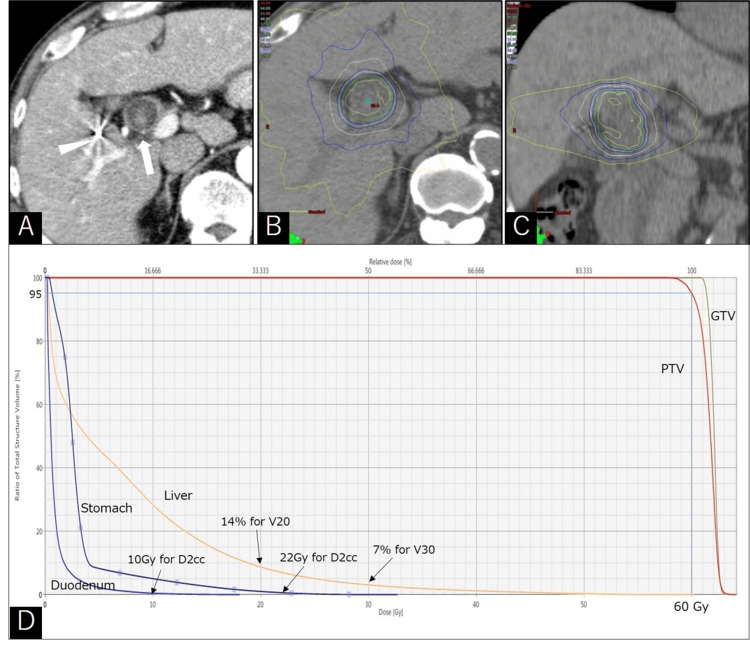
Stereotactic body radiotherapy for intraductal papillary neoplasm of the bile duct PTV: planning target volume; RT: radiation therapy Enhanced CT for RT planning showed the enhanced tumor within the dilated bile duct (arrow) and fiducial gold marker (arrowhead) (A). A dose distribution chart axial view (B) and coronal view (C). The dose-volume histogram for the initial treatment plan shows 60 Gy for 95% of PTV, 10 Gy for D2cc in the duodenum, 22 Gy for D2cc in the stomach, 7% for V30 and 14% for V20 in the liver (D)

Due to difficulty controlling the symptoms of obstructive jaundice and cholangitis with a biliary drainage catheter, a metallic biliary stent was placed; however, it was spontaneously displaced and expelled from the body within one week of placement. As contrast-enhanced computed tomography (CT) of the chest and abdomen was performed and no distant metastases were observed, we considered performing a right hepatectomy; however, as the remnant liver volume after right hepatic lobectomy was estimated to be 40% according to CT volumetric analysis, surgical resection was not considered a viable option in light of the patient’s advanced age and cardiac dysfunction. SBRT was administered as a palliative treatment aimed at controlling the patient’s symptoms, including obstructive jaundice and refractory cholangitis. To prepare for SBRT, a fiducial marker (25G Gold Anchor; Naslund Medical AB, Huddinge, Sweden) was placed percutaneously under ultrasound guidance near the intrabiliary tumor in the right hepatic duct. A planning contrast-enhanced CT scan was obtained during suspended exhalation with the patient in the supine position. According to the planning CT, the contrast-enhanced bile duct tumor was defined as the gross tumor volume. In radiation therapy for cholangiocarcinoma, it is common to create a clinical target volume (CTV) by applying a margin of approximately 20-25 mm along the longitudinal axis of the bile duct to the gross tumor volume. In IPNB, unlike cholangiocarcinoma, the tumor has a low tendency to invade the surrounding tissues; therefore, a margin of 15 mm was applied along the longitudinal axis of the bile duct, and the planning target volume (PTV) was established by adding a 5 mm margin to the CTV in all directions. SBRT was administered during suspended exhalation. Before each irradiation, the positions of the fiducial marker at the time of treatment planning and at the time of irradiation were verified using an onboard imager or cone-beam CT to ensure that the positional error was within 3 mm. The 10-MV X-ray was used for the dose delivery on a Clinac iX linear accelerator (Varian Medical Systems, Palo Alto, CA, USA), and the radiation was delivered using volumetric modulated arc therapy with two arcs. The total dose administered was 60 Gy in eight fractions, with a D95% prescription applied to the PTV. The organs at risk (OARs) were the liver adjacent to the tumor and the duodenum. The V30 of the liver was assessed in 8% of patients, and the D2cc of the duodenum was measured at 12 Gy, both of which were determined to be within the acceptable range (Figure 2).

**Figure 3:**
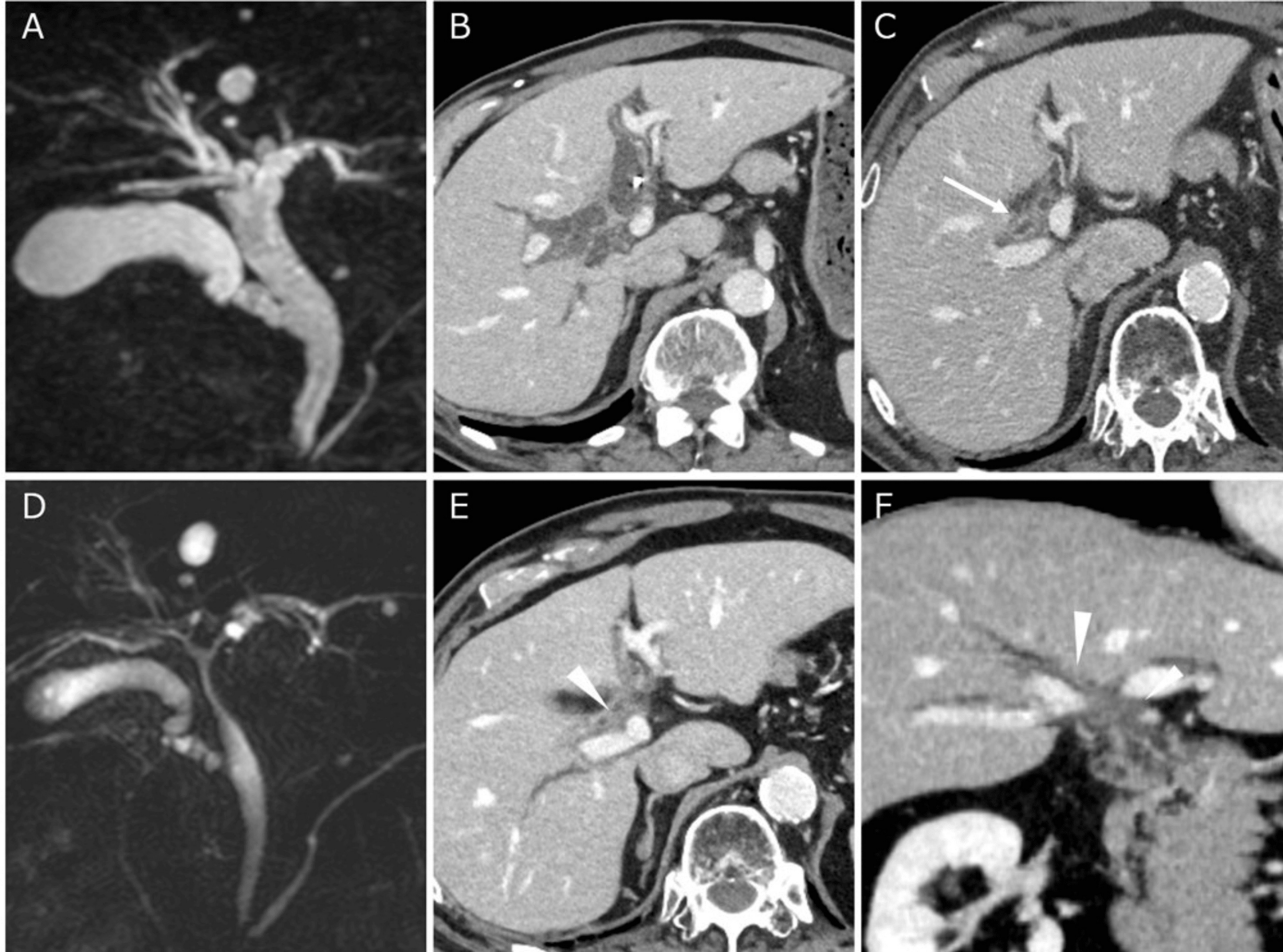
Comparison of MRCP and enhanced CT pre- and post-stereotactic body radiotherapy MRCP: magnetic resonance cholangiopancreatography; CT: computed tomography The biliary duct dilation observed in the pre-treatment MRCP (A) and the contrast-enhanced CT axial image (B). Enhanced CT at the end of radiation therapy showed improvement in biliary duct dilation (arrow). (C). No biliary duct dilation was observed in the MRCP performed at nine months (D) and in the CT scan after one year post-irradiation (arrow head) (E, F).

Upon the completion of SBRT, the refractory abdominal pain and fever observed pre-treatment had improved. Additionally, blood tests performed two weeks post-SBRT showed that the markers associated with cholangitis and jaundice, such as serum bilirubin and C-reactive protein, had normalized. A CT scan obtained one month post-SBRT showed a reduction in the size of the bile duct tumor and an improvement in bile duct dilation, and a CT scan obtained one year later confirmed that the tumor had not grown, nor was a recurrence of the bile duct dilation observed. Additionally, no severe adverse events requiring further treatment, including liver dysfunction or gastrointestinal complications, were observed (Figure 3).

## Discussion

IPNB is a rare tumor occurring in the bile duct and mainly reported in Far Eastern countries, according to the World Health Organization's classification of digestive system tumors [2]. IPNB is defined as the presence of intraductal papillary or villous neoplasms in the dilated bile ducts, covered by neoplastic epithelium and having fine fibrovascular stalks [1,3]. Mucus secretion is frequently observed at the histological and macroscopic levels. Varying degrees of spindle-shaped or cystic bile duct dilatation and multilocular cystic changes are also observed. IPNBs are classified into two types [2]. Type 1 is predominantly observed in East Asia, shares several similarities with intraductal papillary mucinous neoplasms (IPMNs) of the pancreas, and is often associated with mucin production [8-10]. In contrast, type 2 is more frequently observed in Western countries, exhibits less mucin production, and is characterized by a higher degree of cellular atypia. Type 2 carries a higher risk of malignancy than type 1 and is also known to have a higher recurrence rate after surgical resection; therefore, compared to type 2, type 1 IPNBs generally have a better prognosis [3,11,12]. This case showed significant mucin production and, based on the pathological findings, was considered type 1. IPNB is a lesion with a high potential for malignant transformation and is considered a precursor to cholangiocarcinoma. Accordingly, the standard therapeutic approach is surgical resection, which aligns with the guidelines for the management of cholangiocarcinoma [3]. Hepatic lobectomy is often indicated [3,13]; however, being a highly invasive procedure, its application can be challenging, particularly in elderly patients or those with significant cardiovascular comorbidities. In this case, the patient was elderly and ineligible for surgery because of cardiovascular comorbidities. Although systemic chemotherapy is sometimes considered for inoperable cases [4], it is not very effective because there is no established regimen with a high response rate, and in many cases, the patient’s overall condition is poor, making it difficult to introduce systemic therapy. Even in cases where antitumor therapy is not feasible, control of the jaundice and cholangitis caused by high mucus production is necessary, and biliary drainage is commonly performed to relieve these symptoms. However, in cases of jaundice caused by mucin production where no organic biliary stricture is found, biliary stenting for drainage is often ineffective. In the case presented herein, both endoscopic retrograde biliary drainage (ERBD) and metallic stenting were attempted; however, both interventions were ineffective. According to the SEER database, approximately 17.5% of patients diagnosed with IPNB undergo radiotherapy [4], which for IPNB includes palliative irradiation in cases of biliary hemorrhage and high-dose-rate (HDR) brachytherapy using ERBD catheters [6,7]. HDR brachytherapy appears advantageous in regard to dose distribution, particularly with respect to dose reduction to OARs, such as the intestine and liver. Although HDR brachytherapy has this advantage, we opted to perform SBRT instead for two reasons. First, the drainage effect during irradiation is unstable in patients with IPNB, and the diameter of the bile duct is likely to change significantly during irradiation, making dose evaluation difficult. Second, there was a limited number of facilities where HDR brachytherapy could be performed, making it difficult for elderly patients to get to the appropriate facility. SBRT for intrahepatic cholangiocarcinoma has been reported to achieve high local control rates with a biologically effective dose (BED) of 100 Gy or higher. In this case, treatment was administered with a dose of 60 Gy across eight fractions (BED, 105 Gy; EQD2 = 87.5 Gy; α/β = 10), following a standard radiation dose for intrahepatic cholangiocarcinoma; therefore, the high dose of radiation may have contributed not only to the suppression of mucin production but also to favorable local tumor control [14-17]. Gastrointestinal complications are significant adverse events associated with SBRT for the treatment of biliary tumors. A report showed that patients receiving a dose exceeding an EQD2 of 50 Gy to the intestine have a 2%-9% incidence of late-onset obstructive small bowel ileus or perforation [18]. Therefore, precise irradiation with minimal margins is essential to safely deliver the higher doses required for SBRT. In this case, gold fiducial markers were placed in the region near the tumor to facilitate accurate targeting, and daily verification was performed using onboard imaging to confirm the position of the marker before each treatment. Additionally, respiratory training was repeatedly conducted to reduce tumor motion due to breathing. In this case, the duodenal dose was limited to an EQD2 of 38 Gy at D2cc, and the dose to the small intestine did not exceed 50 Gy. Regarding the safety of SBRT for the hepatic hilum, a report indicated that biliary complications may occur in cases with a large high-dose irradiation area or those with a metallic stent [19]. However, there are also reports suggesting that irradiation can be performed relatively safely at a prescribed dose similar to that used in this case [20]. In our institution's experience, we have not encountered cases of severe biliary complications following irradiation to the hepatic hilum, and we consider SBRT to be a relatively safe treatment option in this region. One year after radiation, no severe adverse gastrointestinal events were observed. These results suggest that administering SBRT for the management of inoperable IPNB with mucin secretion not only suppresses mucin secretion and facilitates improvement of jaundice and cholangitis but also exerts an antitumor effect and may prevent future progression to cholangiocarcinoma. The accumulation of additional cases would suggest that SBRT may be a promising treatment option for unresectable IPNB.

## Conclusions

We have presented a case herein of SBRT performed for the management of an inoperable IPNB, in which good local control and symptomatic relief were obtained without serious adverse events. To the best of our knowledge, this is the first report of a case of unresectable IPNB treated with SBRT using a definitive radiation dose. Further case reports are needed to determine the optimal irradiation method and dose fractionation of SBRT for the treatment of IPNB and to evaluate the safety of administering SBRT in this manner.
